# Sensitivity of magnetic resonance tomographic angiography for detecting the degree of neurovascular compression in trigeminal neuralgia

**DOI:** 10.1007/s10072-020-04419-0

**Published:** 2020-04-29

**Authors:** Yun-bo Hao, Wei-jie Zhang, Min-jie Chen, Ying Chai, Wen-hao Zhang, Wen-bin Wei

**Affiliations:** Department of Oral and Maxillofacial Surgery, College of Stomatology, Ninth People’s Hospital, Shanghai Jiao Tong University School of Medicine, Shanghai, China

**Keywords:** Magnetic resonance tomographic angiography, Microvascular decompression, Endoscope, Trigeminal neuralgia

## Abstract

**Purpose:**

Neurovascular compression (NVC) is hypothesized to be the main pathogenic factor of trigeminal neuralgia (TN). Microvascular decompression (MVD) has become a popular surgery for TN, and the success rate depends on the degree of NVC. As the routine examination before MVD, magnetic resonance tomographic angiography (MRTA) shows high sensitivity for detecting NVC. However, there are no reports on the sensitivity of MRTA for assessing the degree of NVC.

**Methods:**

This study aimed to evaluate the sensitivity of MRTA for determining the degree of NVC by comparing preoperative MRTA and intraoperative endoscopy findings. A total of 480 patients who suffered from TN and underwent MVD were included. Their preoperative MRTA and intraoperative endoscopy findings were reviewed. The kappa test was used to identify similarities between the MRTA and endoscopy findings.

**Results:**

The degree of NVC on preoperative MRTA was similar to that on endoscopy (kappa = 0.770). The number of offending vessels according to preoperative MRTA was coincident with that according to endoscopy (kappa = 0.722).

**Conclusion:**

MRTA had high sensitivity for detecting not only the presence of NVC but also the degree of NVC.

Trigeminal neuralgia (TN) is one of the most common facial pain disorders. Vascular compression is a widely acknowledged theory for the pathogenesis of TN. Neurovascular compression (NVC) was first reported by Gardner and Miklos [1] and is defined as direct contact between vessels and nerves. Microvascular decompression (MVD), which is now the standard surgery for TN, is based on the theory of NVC [2]. The surgery can efficiently and safely cure TN and reduce the possibility of facial numbness [3].

The success rate of MVD depends on the correct clinical indications and the preoperative radiological examination of NVC. Among all imaging modalities, magnetic resonance tomographic angiography (MRTA) has been accepted as the conventional radiological evaluation to perform before MVD. Commonly, compression by vessels or tumors occurs in the cerebellopontine angle (CPA) [4], and the vessel that most commonly causes TN is the superior cerebellar artery [5]. In many studies, the radiological indication for MVD was the presence of NVC on MRTA, but the degree of compression was not considered. Recently, some articles have indicated that the more severe the compression is, the better the effect of MVD [6]. However, the degree of compression can only be determined intraoperatively. In recent articles, only the presence of NVC on MRTA has been reported. However, assessments of the degree of NVC have not been reported.

The purpose of this study was to evaluate the accuracy of the degree of NVC determined on MRTA by comparing the findings of MRTA with those of endoscopy during MVD.

## Methods

### Patients

A total of 480 patients with classical TN underwent endoscopy-assisted MVD at the Shanghai Ninth People’s Hospital, Shanghai Jiao Tong University Medical School, from May 2003 to July 2019. A total of 293 women and 187 men underwent surgery, and their ages ranged from 19 to 85. There were 180 cases of NVC on the left side and 300 on the right side. All patients underwent MRTA before the operation, and imaging revealed no abnormalities, such as multiple sclerosis, vascular malformations, or tumors. In all patients, the pain was uncontrollable with medicine. All patients were operated on by two senior neurosurgeons (Wei-jie Zhang and Min-jie Chen) with the same microsurgical technique.

### MRTA technique

All patients underwent MRTA with a 3.0-T MRI system (Signa 3.0 T Twinspeed; GE, Fairfield, CT) and the following parameters: repetition time (TR), 25.0 ms; echo time (TE), 3.8 ms; flip angle, 20°; matrix size, 256 × 160; number of acquisitions, 1; volume size, 20 mm; and bandwidth, 11.90. The slice thickness was 1 mm. The images were obtained in 3 standard planes: coronal, sagittal, and axial.

### Evaluation of the degree of NVC on MRTA

**Fig. 1:**
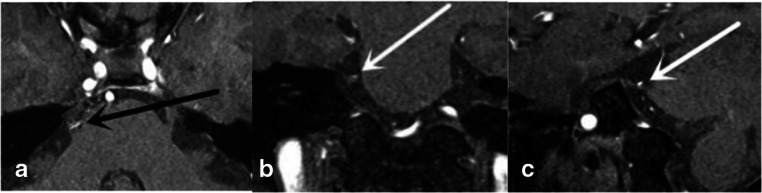
Degree of NVC in one patient. Image **a** was scored as 2 points. Image **b** was scored as 2 points. Image **c** was scored as 2 points. The total score was 6 points, and the grade was II

Two neurosurgeons evaluated the MRTA images separately. The classification of the degree of NVC was based on Chen’s report [7]: 0′, there was no relationship between the nerve and the vessel, or the relationship was vague and difficult to evaluate; 1′, the vessel crossed or touched the nerve without a visible layer of cerebrospinal fluid (CSF) and without any deformity of the root; 2′, a marked indentation was present on the root, caused by compression from the offending vessel; and 3′, distortion and/or displacement of the root, compared with the asymptomatic side, was observed. Three separate scores from the axial, oblique sagittal, and coronal images were added together. The severity of the compression was classified as follows: grade 0, the total score was 0 to 1; grade 1, the total score was from 2 to 3; grade 2, the total score was from 4 to 6; and grade 3, the total score was from 7 to 9 (Fig. 1, Table 1).

**Table 1:** Evaluation of MRTA and surgical findings

	Grade 0	Grade 1	Grade 2	Grade 3
MRTA findings	0–1 point	2–3 points	4–6 points	7–9 points
Surgical findings	No or blurred relationships	Vessels crossing or in contact with the nerve	Indentation on the nerve	Severe displacement of the nerve

### Evaluation of the degree of NVC on endoscopy during MVD

**Fig. 2:**
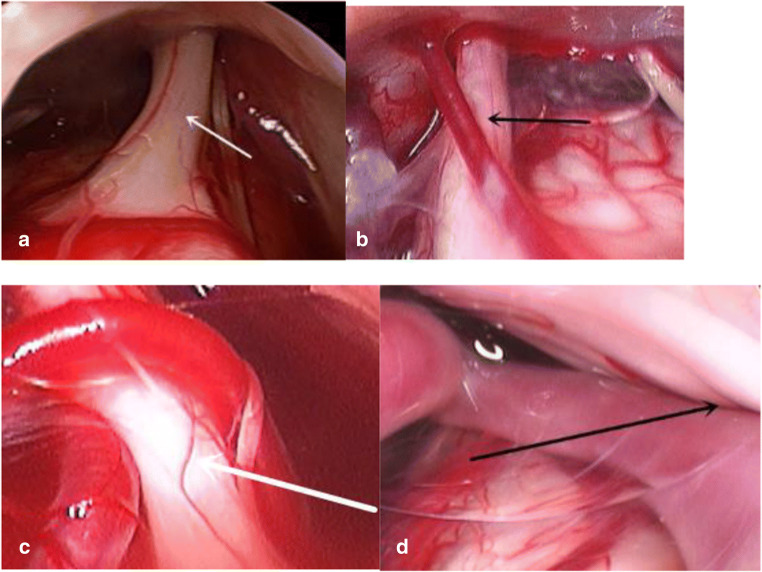
Classification of the degree of NVC on endoscopy. **a** Grade 0. **b** Grade 1. **c** Grade 2. **d** Grade 3. Arrows indicate the trigeminal nerve

Two senior neurosurgeons separately evaluated the endoscopic images after MVD. NVC was graded as follows on endoscopy [8, 9]: grade 0, there was no relationship between the nerve and vessels; grade 1, there was contact between the nerve and vessels without evidence of compression; grade 2, a groove had formed on the nerve from vascular compression; and grade 3, the nerve has been distorted by vascular compression (Fig. 2, Table 1).

### Statistical analysis

We evaluated the consistency between the MRTA and surgical findings with the kappa test [10]. The agreement was considered “poor” if the kappa coefficient was 0–0.19, “fair” if the kappa coefficient was 0.20–0.39, “moderate” if the kappa coefficient was 0.40–0.59, “substantial” if the kappa coefficient was 0.60–0.79, and “almost perfect” if the kappa coefficient was 0.80–1.00 (*p* < 0.05).

## Results

### Sensitivity and specificity of MRTA

On MRTA, 439 (91.5%, 439/480) patients had offending vessels on the symptomatic side, and 41 (8.5%, 41/480) were diagnosed without offending vessels. Regarding the surgical findings, 449 (93.5%, 449/480) patients had offending vessels, and 31 (6.5%, 31/480) were diagnosed without offending vessels.

Among the 449 patients who had NVC on endoscopy, 430 patients were also diagnosed on MRTA (Table 2). Among the 31 patients without NVC on endoscopy, 22 patients were diagnosed on MRTA (Table 2). The sensitivity of MRTA was 95.8% (430/449), and the specificity was 71.0% (22/31).

**Table 2:** Sensitivity and specificity of MRTA

MRTA findings	Surgical findings		Total
	NVC+	NVC−	
NVC+	430	9	439
NVC−	19	22	41
Total	449	31	480

### Degree of NVC on MRTA and endoscopy

According to our classification, the severity of NVC on MRTA and endoscopy was classified into 4 grades (Table 3). On MRTA, 41 (8.5%, 41/480) patients were grade 0, 110 (22.9%, 110/480) patients were grade 1, 252 (52.5%, 252/480) patients were grade 2, and 77 (16.1%, 77/480) patients were grade 3. On endoscopy, 31 (6.5%, 31/480) patients were grade 0, 133 (27.7%, 133/480) patients were grade 1, 241 (50.2%, 241/480) patients were grade 2, and 75 (15.6%, 75/480) patients were grade 3. There was strong consistency between the preoperative MRTA and intraoperative endoscopy findings (kappa = 0.770).

**Table 3:** Grade according to MRTA and surgical findings

MRTA findings	Surgical findings				Total
	Grade 0	Grade 1	Grade 2	Grade 3	
Grade 0	22	17	0	2	41
Grade 1	6	96	8	0	110
Grade 2	3	19	224	6	252
Grade 3	0	1	9	67	77
Total	31	133	241	75	480

### Number of compressed vessels

We counted the number of offending vessels on preoperative MRTA and intraoperative endoscopy (Table 4). Endoscopy showed 31 (6.5%, 31/480) patients without NVC, 372 (77.5%, 372/480) patients with 1 offending vessel, 70 (14.6%, 70/480) patients with 2 offending vessels, and 7 (1.4%, 7/480) patients with 3 offending vessels. MRTA showed 41 (8.6%, 41/480) patients with no NVC, 367 (76.5%, 367/480) patients with one offending vessel, 65 (13.5%, 65/480) patients with 2 offending vessels, and 7 (1.4%, 7/480) patients with 3 offending vessels. There was consistency between the preoperative MRTA and intraoperative endoscopy findings (kappa = 0.722).

**Table 4:** Number of offending vessels according to MRTA and surgical findings

MRTA findings	Surgical findings				Total
	0	1	2	3	
0	22	19	0	0	41
1	8	345	13	1	367
2	1	8	56	0	65
3	0	0	1	6	7
Total	31	372	70	7	480

## Discussion

TN is a disease that greatly impacts quality of life. The most commonly and widely acknowledged reason for TN is NVC. As it can address the possible causes of TN, MVD [11] is an effective treatment for classical TN and carries a lower risk of nerve damage than other treatments, such as percutaneous radiofrequency thermocoagulation and X-ray knife surgery [3, 12]. Generally, trigeminal nerve damage can cause numbness in the area controlled by the nerve. Therefore, surgical treatment is the ideal option for TN if permitted by the patient’s physical condition. To confirm TN, all patients need to undergo preoperative MRTA.

MRTA was developed based on 3D time-of-flight magnetic resonance angiography (TOF-MRA) sequences. With this technique, high-speed blood flow presents a high signal intensity, while CSF presents a low signal intensity, and nerves present an intermediate signal intensity. MRTA increases the contrast between vessels and nerves, so it can be used to obtain high-resolution images of NVC around the CPA [13] with higher sensitivity (88–96.7%) [14–19] and higher specificity (50–100%) than TOF-MRA [15–19]. In our study, we used MRTA images on three planes to help confirm NVC. The sensitivity of MRTA was 95.8%, and the specificity was 71.0%. These results are similar to those previously published.

The effects of MVD are greatly dependent on the degree of NVC. Kwang et al. [6] reported that the efficiency of MVD for patients with grade 0 or 1 disease was only 33.3% and was 0% at 5 years, while the success rate of MVD for grade 2 disease was 80.3% and that for grade 3 disease was 96.4%. Paulo et al. [15] reported that the success rate at 15 years was 88.1% for grade 3 NVC, 78.3% for grade 2 NVC, and only 58.3% for grade 1 NVC. The ability to predict the success rate is necessary when selecting a treatment. Thus, MVD is recommended for patients with grade 2 or 3 NVC on preoperative MRTA, as MVD is the best choice for these patients. However, for patients with grade 0 or 1 disease, especially elderly patients with chronic diseases, percutaneous radiofrequency thermocoagulation, balloon compression, or radiosurgery could be preferred.

Previously, the degree of NVC was determined during MVD. The more severe the NVC found during MVD was, the higher the success rate. In most preoperative MRTA evaluations, close attention is paid to the presence of NVC, and the reported sensitivities and specificities were for detecting the presence of NVC. The accuracy of MRTA in determining the degree of NVC was not considered.

In our study, the degree of NVC could be preoperatively assessed on MRTA through our classification system. The endoscopy findings were considered the gold standard because of the wide visual field of endoscopy. The degree of NVC determined on MRTA was compared with that determined on endoscopy, and there was a high degree of consistency between the results (kappa = 0.770).

Additionally, the number of offending vessels was compared, and consistency was found between the preoperative MRTA and intraoperative endoscopy findings (kappa = 0.722). In our study, the number of offending vessels identified on endoscopy was 0 in 6.5% of the patients, 1 in 77.5% of the patients, 2 in 14.6% of the patients, and 3 in 1.4% of the patients. However, on MRTA, the correct number of offending vessels was sometimes unable to be determined. Some articles have reported that veins, to some extent, may cause TN [20], and it was difficult to find veins on MRTA. One explanation for this could be the lower sensitivity of MRTA for detecting veins with a slow flow velocity [21, 22].

## Conclusion

MRTA could predict not only the presence of NVC but also the degree of NVC and the number of offending vessels. This could help surgeons select the best surgical option.

## Abbreviations

NVC: Neurovascular compression
TN: Trigeminal neuralgia
MVD: Microvascular decompression
MRTA: Magnetic resonance tomographic angiography
CPA: Cerebellopontine angle

## References

[1] Gardner WJ, Miklos MV (1959). Response of trigeminal neuralgia to decompression of sensory root; discussion of cause of trigeminal neuralgia. J Am Med Assoc.

[2] Zhong J, Zhu J, Sun H, Dou NN, Wang YN, Ying TT, Xia L, Liu MX, Tao BB, Li ST (2014). Microvascular decompression surgery: surgical principles and technical nuances based on 4000 cases. Neurol Res.

[3] Barker FG, Jannetta PJ, Bissonette DJ, Larkins MV, Jho HD (1996). The long-term outcome of microvascular decompression for trigeminal neuralgia. J N Engl J Med.

[4] Jito J, Nozaki K (2016). Trigeminal neuralgia attributable to intraneural trigeminocerebellar artery: case report and review of the literature. World Neurosurg.

[5] Sarsam Z, Garcia-Fiñana M, Nurmikko TJ, Varma TRK, Eldridge P (2010). The long-term outcome of microvascular decompression for trigeminal neuralgia. Br J Neurosurg.

[6] Lorenzoni J, David P, Levivier M (2012). Patterns of neurovascular compression in patients with classic trigeminal neuralgia: a high-resolution MRI-based study. Eur J Radiol.

[7] Chai Y, Chen M, Zhang W, Zhang W (2013). Predicting the outcome of microvascular decompression for primary trigeminal neuralgia by the use of magnetic resonance tomographic angiography. J Craniofac Surg.

[8] Chen MJ, Zhang WJ, Yang C, Wu YQ, Zhang ZY, Wang Y (2008). Endoscopic neurovascular perspective in microvascular decompression of trigeminal neuralgia. J Craniomaxillofac Surg.

[9] Berk C (2001). Bilateral trigeminal neuralgia: a therapeutic dilemma. Br J Neurosurg.

[10] Sindou M, Leston J, Decullier E, Chapuis F (2007). Microvascular decompression for primary trigeminal neuralgia: long-term effectiveness and prognostic factors in a series of 362 consecutive patients with clear-cut neurovascular conflicts who underwent pure decompression. J Neurosurg.

[11] Meaney JF, Eldridge PR, Dunn LT, Nixon TE, Whitehouse GH, Miles JB (1995). Demonstration of neurovascular compression in trigeminal neuralgia with magnetic resonance imaging. Comparison with surgical findings in 52 consecutive operative cases. J Neurosurg.

[12] Xia L, Zhong J, Zhu J, Wang YN, Dou NN, Liu MX, Visocchi M, Li ST (2014). Effectiveness and safety of microvascular decompression surgery for treatment of trigeminal neuralgia: a systematic review. J Craniofac Surg.

[13] Landis JR, Koch GG (1977). The measurement of observer agreement for categorical data. Biometrics.

[14] Anderson VC, Berryhill PC, Sandquist MA, Ciaverella DP, Nesbit GM, Burchiel KJ (2006). High-resolution three-dimensional magnetic resonance angiography and three-dimensional spoiled gradient-recalled imaging in the evaluation of neurovascular compression in patients with trigeminal neuralgia: a double-blind pilot study. Neurosurgery.

[15] Leal PR, Hermier M, Froment JC, Souza MA, Cristino-Filho G, Sindou M (2010). Preoperative demonstration of the neurovascular compression characteristics with special emphasis on the degree of compression, using high-resolution magnetic resonance imaging: a prospective study, with comparison to surgical findings, in 100 consecutive patients who underwent microvascular decompression for trigeminal neuralgia. Acta Neurochir.

[16] Jo KW, Kong DS, Hong KS, Lee JA, Park K (2013). Long-term prognostic factors for microvascular decompression for trigeminal neuralgia. J Clin Neurosci.

[17] Zhang W, Chen M, Zhang W, Chai Y (2014). Etiologic exploration of magnetic resonance tomographic angiography negative trigeminal neuralgia. J Clin Neurosci.

[18] Leal PR, Froment JC, Sindou M (2009). Predictive value of MRI for detecting and characterizing vascular compression in cranial nerve hyperactivity syndromes (trigeminal and facial nerves). Neurochirurgie.

[19] Boecher-Schwarz HG, Bruehl K, Kessel G, Guenthner M, Perneczky A, Stoeter P (1998). Sensitivity and specificity of MRA in the diagnosis of neurovascular compression in patients with trigeminal neuralgia. Neuroradiology.

[20] Chen MJ, Zhang WJ, Guo ZL, Yang C, Zhang WH, Dong MJ, Chai Y, Zhang ZY (2014). Preoperative evaluation of the neurovascular compression using magnetic resonance tomographic angiography: our radiologic indications for microvascular decompression to treat trigeminal neuralgia. J Craniofac Surg.

[21] Helbig GM, Callahan JD, Cohen-Gadol AA (2009). Variant intraneural vein-trigeminal nerve relationships: an observation during microvascular decompression surgery for trigeminal neuralgia. Neurosurgery.

[22] Umehara F, Kamishima K, Kashio N, Yamaguchi K, Sakimoto T, Osame M (1995). Magnetic resonance tomographic angiography: diagnostic value in trigeminal neuralgia. Neuroradiology.

